# *De Novo* Assembly of the Northern Cardinal (*Cardinalis cardinalis*) Genome Reveals Candidate Regulatory Regions for Sexually Dichromatic Red Plumage Coloration

**DOI:** 10.1534/g3.120.401373

**Published:** 2020-08-13

**Authors:** Simon Yung Wa Sin, Lily Lu, Scott V. Edwards

**Affiliations:** * Department of Organismic and Evolutionary Biology, Museum of Comparative Zoology, Harvard University, 26 Oxford Street, Cambridge, MA 02138; † School of Biological Sciences, The University of Hong Kong, Pok Fu Lam Road, Hong Kong

**Keywords:** AllPaths-LG, *Cis*-regulatory elements, *CYP2J19* gene, Ketocarotenoid pigments, Transcription factors

## Abstract

Northern cardinals (*Cardinalis cardinalis*) are common, mid-sized passerines widely distributed in North America. As an iconic species with strong sexual dichromatism, it has been the focus of extensive ecological and evolutionary research, yet genomic studies investigating the evolution of genotype–phenotype association of plumage coloration and dichromatism are lacking. Here we present a new, highly-contiguous assembly for *C. cardinalis*. We generated a 1.1 Gb assembly comprised of 4,762 scaffolds, with a scaffold N50 of 3.6 Mb, a contig N50 of 114.4 kb and a longest scaffold of 19.7 Mb. We identified 93.5% complete and single-copy orthologs from an Aves dataset using BUSCO, demonstrating high completeness of the genome assembly. We annotated the genomic region comprising the *CYP2J19* gene, which plays a pivotal role in the red coloration in birds. Comparative analyses demonstrated non-exonic regions unique to the *CYP2J19* gene in passerines and a long insertion upstream of the gene in *C. cardinalis*. Transcription factor binding motifs discovered in the unique insertion region in *C. cardinalis* suggest potential androgen-regulated mechanisms underlying sexual dichromatism. Pairwise Sequential Markovian Coalescent (PSMC) analysis of the genome reveals fluctuations in historic effective population size between 100,000–250,000 in the last 2 millions years, with declines concordant with the beginning of the Pleistocene epoch and Last Glacial Period. This draft genome of *C. cardinalis* provides an important resource for future studies of ecological, evolutionary, and functional genomics in cardinals and other birds.

The northern cardinal (*Cardinalis cardinalis*) is a mid-sized (∼42-48 g) passerine broadly distributed in eastern and central North America, with a range encompassing northern Central America to southeastern Canada (Smith *et al.* 2011). It has high genetic (Smith *et al.* 2011) and phenotypic (Halkin and Linville 2020) diversity and is currently divided into 18 subspecies (Paynter and Storer 1970). To explain the pattern of high genetic diversity and subspecies divergence in this species, the ecology and phylogeography should be considered together with accurate inference of demographic history, which is facilitated by the availability of whole-genome data. Cardinals have also been studied extensively on research areas such as song and communication (*e.g.*, Jawor and MacDougall-Shackleton 2008; Anderson and Conner 1985; Yamaguchi 1998), sexual selection (*e.g.*, Jawor *et al.* 2003), physiology (*e.g.*, DeVries and Jawor 2013; Wright and Fokidis 2016), and plumage coloration (*e.g.*, Linville and Breitwisch 1997; Linville *et al.* 1998; McGraw *et al.* 2003).

*C. cardinalis* is an iconic species in the Cardinalidae family and has strong sexual dichromatism, with adult males possessing bright red plumage and adult females tan (Figure 1). Many other species in Cardinalidae are also sexually dichromatic with the male being partly red or completely red like *C. cardinalis* and the female drab in color. Examples are other species in *Cardinalis*, all species in *Piranga* including the scarlet tanager (*P. olivacea*) and summer tanager (*P. rubra*), and some species in *Habia*, *Granatellus* and *Caryothraustes*. The red plumage coloration of many bird species plays important roles in social and sexual signaling, and research on this trait has deepened our understanding of sexual selection. With the advance of genomic technologies in the last several years, the genetic basis and evolution of plumage coloration in birds is under intense investigation (Lopes *et al.* 2016; Mundy *et al.* 2016; Funk and Taylor 2019; Kim *et al.* 2019; Toomey *et al.* 2018; Twyman *et al.* 2018a; Twyman *et al.* 2016; Twyman *et al.* 2018b; Gazda *et al.* 2020b; Aguillon *et al.* 2020). The red color of feathers is generated by the deposition of ingested carotenoids, modified endogenously. A ketolase is involved in an oxidative reaction to convert dietary yellow carotenoids into red ketocarotenoids (Friedman *et al.* 2014). Recently the gene encoding the carotenoid ketolase has been shown to be a cytochrome P450 enzyme, *CYP2J19* (Lopes *et al.* 2016; Mundy *et al.* 2016). The identification of *CYP2J19* as the gene responsible for red coloration in hybrid canary (*Serinus canaria*) plumage (Lopes *et al.* 2016) and zebra finch (*Taeniopygia guttata*) bill and legs (Mundy *et al.* 2016) suggests its general role in red pigmentation of multiple tissues across birds. *C. cardinalis* is an excellent candidate to further our understanding of the regulation and development of red plumage coloration in birds. In particular, little attention has been paid to noncoding, regulatory signatures in the region surrounding *CYP2J19*, and genomic resources for cardinals would greatly facilitate such work.

**Figure 1:**
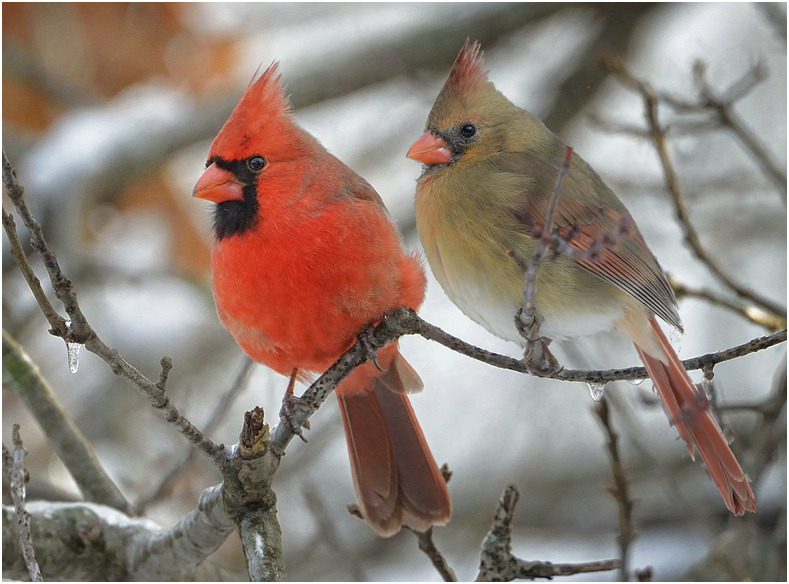
A pair of northern cardinals, a common, sexually dichromatic passerine bird. The adult male (left) has bright red plumage whereas the adult female (right) is primarily tan in color. Photo © Clarence Stewart.

Despite extensive ecological and evolutionary research on *C. cardinalis*, we lack a highly contiguous genome of this species and other species in the family Cardinalidae. A genome assembly of *C. cardinalis* will facilitate studies of genotype–phenotype association of sexually dichromatic plumage color in this species, and facilitate comparative genomic analysis in birds generally. Here we generate a new *de novo* genome assembly from a male collected in Texas, USA, accompanied a voucher specimen in the ornithology collection of the Museum of Comparative Zoology, Harvard University, MA, USA. We also performed comparative genomic analyses of the *CYP2J19* region to identify potential non-coding regulatory regions and demographic analysis to examine the fluctuation in historic effective population size. Given the lack of genomic resources currently available for the Cardinalidae, this *C. cardinalis* draft genome and our new findings will facilitate future genomic studies of cardinals and other passerines.

## Methods & Materials

### Sample collection and DNA extraction

We collected a male *C. cardinalis* on the DNA Works Ranch, Afton, Dickens County, Texas, United States (33.76286°, -100.8168°) on January 19, 2016 (MCZ Ornithology cat. no.: 364215). We collected muscle, heart, and liver and flash froze the tissues in liquid nitrogen immediately in the field. Upon returning from the field, tissues were stored at −196° in the cryopreservation facility of the Museum of Comparative Zoology until DNA extraction. We isolated genomic DNA using the DNeasy Blood and Tissue Kit (Qiagen, Hilden, Germany) following the manufacturer’s protocol. We confirmed the sex of the individual using published PCR primers targeting *CHD1* genes with different sizes of intron on the W and Z chromosomes (2550F & 2718R; Fridolfsson and Ellegren 1999) and measured DNA concentration with a Qubit dsDNA HS Assay Kit (Invitrogen, Carlsbad, USA).

### Library preparation and sequencing

We performed whole-genome library preparation and sequencing following Grayson *et al.* (2017). In brief, a DNA fragment library of 220 bp insert size was prepared using the PrepX ILM 32i DNA Library Kit (Takara), and mate-pair libraries of 3 kb insert size were prepared using the Nextera Mate Pair Sample Preparation Kit (cat. No. FC-132-1001, Illumina). We performed DNA shearing for the fragment and mate-pair library preparations using Covaris S220. We used the 0.75% agarose cassette in the Pippin Prep (Sage Science) for size selection of the mate-pair library (target size 3 kb, “Tight” mode). We then assessed fragment and mate-pair library qualities using the High Sensitivity D1000 ScreenTape for the Tapestation (Agilent) and High Sensitivity DNA Kit for the Bioanalyzer (Agilent), respectively, and quantified the libraries with qPCR (KAPA library quantification kit) prior to sequencing. We sequenced the fragment and mate-pair libraries on an Illumina HiSeq 2500 instrument (High Output 250 kit, PE 125 bp reads) at the Bauer Core facility at Harvard University for 0.78 and 0.72 lane, respectively.

### De novo genome assembly and assessment

We assessed the quality of the sequencing data using FastQC and removed adapters using Trimmomatic (Bolger *et al.* 2014). We assembled the genome using AllPaths-LG v52488 (Gnerre *et al.* 2011), which allowed us to estimate the genome size from k-mer frequencies and assess the contiguity of the *de novo* genome. We provided the standard deviation as 10% of the library insert size for the genome assembly setting. We estimated the completeness of the assembled genome with BUSCO v2.0 (Simão *et al.* 2015) and used the aves_odb9 dataset to search for 4915 universal single-copy orthologs in birds.

### Analysis of the CYP2J19 genomic region

We annotated the genomic region that comprises the *CYP2J19* gene using blastn with the *CYP2J19*, *CYP2J40*, *HOOK1* and *NFIA* genes from *S. canaria* and *T. guttata* (gene location: chromosome 8, NC_045007.1 (3177374..3202478)) as queries. Conserved non-exonic elements (CNEEs) were obtained from a published UCSC Genome Browser track hub containing a progressiveCactus alignment of 42 bird and reptile species and CNEE annotations (viewed in the UCSC genome browser at https://ifx.rc.fas.harvard.edu/pub/ratiteHub8/hub.txt; Sackton *et al.* 2019). We identified *CYP2J19* genes from 30 bird species (Twyman *et al.* 2018a) by querying their genome assemblies from the NCBI database using BLAST. The alignment was compiled using MUSCLE (Edgar 2004) and viewed with Geneious (Kearse *et al.* 2012). We also aligned the genomic regions up- and downstream of *CYP2J19* in 10 passerines to identify any potential conserved regulatory regions present in species with red carotenoid coloration.

We used the MEME Suite (Bailey *et al.* 2009; Bailey *et al.* 2015) to discover potential regulatory DNA motifs in the region upstream of the *CYP2J19* gene that we found to be unique to *C. cardinalis* and to predict transcription factors (TFs) binding to those motifs. We used MEME (Bailey and Elkan 1994) to identify the top five most statistically significant motifs and their corresponding positions in the region. We used Tomtom (Gupta *et al.* 2007) to compare the identified motifs against databases (*e.g.*, JASPAR) of known motifs and identify potential TFs specific to those matched motifs. To search for potential biological roles of these motifs, we used GOMo (Buske *et al.* 2010) to scan all promoters in *Gallus gallus* using the identified motifs to determine if any motifs are significantly associated with genes linked to any Genome Ontology (GO) terms.

### Inference of demographic history

To investigate historical demographic changes in the northern cardinal we used the Pairwise Sequential Markovian Coalescent (PSMC) model (Li and Durbin 2011) based on the diploid whole-genome sequence to reconstruct the population history. We generated consensus sequences for all autosomes using SAMtools’ (v1.5; Li *et al.* 2009) *mpileup* command and the *vcf2fq* command from vcfutils.pl. We applied filters for base quality and mapping quality below 30. The settings for the PSMC atomic time intervals were “4+30*2+4+6+10”. 100 bootstraps were used to compute the variance in estimates of *N_e_*. To convert inferred population sizes and times to numbers of individuals and years, respectively, we used the estimate of mutation rate of 3.44e-09 per site per generation from the medium ground finch (*Geospiza fortis*, Thraupidae, a closely related passerine clade) (Nadachowska-Brzyska *et al.* 2015). We estimated the generation time of the northern cardinal as age of sexual maturity multiplied by a factor of two (Nadachowska-Brzyska *et al.* 2015), yielding a generation time of 2 years.

### Data availability

Data from the final genome assembly is available from NCBI (BioProject number: PRJNA642398; BioSample number: SAMN15394674; Accession number: JACDOX000000000). Supplemental material available at figshare: https://doi.org/10.25387/g3.12798521.

## Results and Discussion

### Genome assembly and evaluation

We generated 703,754,970 total reads from two different sequencing libraries: 335,713,090 reads from the fragment library and 368,041,880 reads from the mate-pair library. The genome size estimated by AllPaths-LG from k-mers was 1.1 Gb (Table 1). The estimated sequence coverage was ∼59x. The assembly consisted of 32,783 contigs placed in 4,762 scaffolds. The largest scaffold was 19.7 Mb. The contig N50 was 114.4 kb and the scaffold N50 was 3.6 Mb (Table 1). The number of contigs per Mb was 31.4 and the number of scaffolds per Mb was 4.6. The average GC content of the assembly was 42.1%. BUSCO scores (Simão *et al.* 2015) suggest high completeness of the genome, with 93.5% of single-copy orthologs for birds identified (Table 2). The genome contiguity is among the best genomes (Fig. S1) presented in Jarvis *et al.* (2014) and Grayson *et al.* (2017), and is better than the *C. cardinalis* genome assembled in Feng *et al*. (in press) in terms of both contig N50 and scaffold N50.

**Table 1:** *De novo* assembly metrics for northern cardinal genome

Metric	Value
Estimated genome size	1.10 Gb
%GC content	42.1
Total depth of coverage	59x
Total contig length (bp)	1,019,501,986
Total scaffold length (bp, gapped)	1,044,184,327
Number of contigs	32,783
Contig N50	114.4 kb
Number of scaffolds	4,762
Scaffold N50 (with gaps)	3.6 Mb
Largest scaffold	19.7 Mb

**Table 2:** Output from BUSCO analyses to assess genome completeness by searching for single-copy orthologs from aves dataset

	Aves	%
Complete BUSCOs	4642	94.4
Complete and single-copy BUSCOs	4596	93.5
Complete and duplicated BUSCOs	46	0.9
Fragmented BUSCOs	167	3.4
Missing BUSCOs	106	2.2
Total BUSCO groups searched	4915	

### Candidate non-coding regulatory regions of CYP2J19

**Figure 2:**
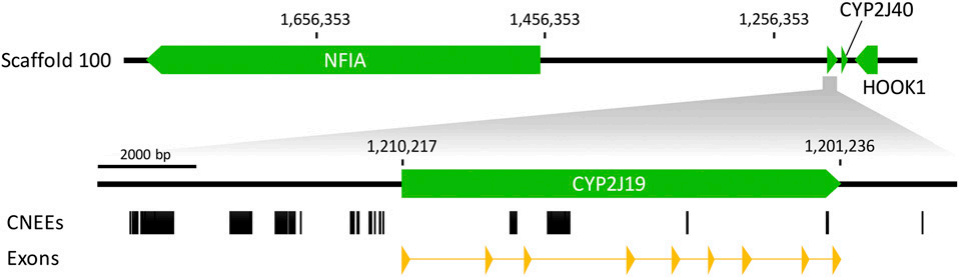
Annotation of the *CYP2J19* region in scaffold 100 of the northern cardinal genome. The *CYP2J19* gene is flanked by the *NFIA* and *CYP2J40* genes as in other passerines. Conserved non-exonic elements (CNEEs) are shown in black boxes. Exons of the *CYP2J19* gene are in yellow triangles.

The *CYP2J19* gene was identified on scaffold 100 of the *C. cardinalis* genome, flanked by *CYP2J40* and *NFIA* genes (Figure 2), an arrangement consistent with other species (*e.g.*, Lopes *et al.* 2016; Mundy *et al.* 2016). The length of the *CYP2J19* gene in *C. cardinalis* is 8982 bp, comprising 9 exons. We identified 7 CNEEs in the *CYP2J19* gene, 24 CNEEs within ∼6 kb upstream and 1 CNEE within ∼6 kb downstream of the gene. Of those 32 CNEEs, 4 CNEEs in *CYP2J19* and 7 CNEEs upstream of the gene are at least 50 bp in length.

**Figure 3:**
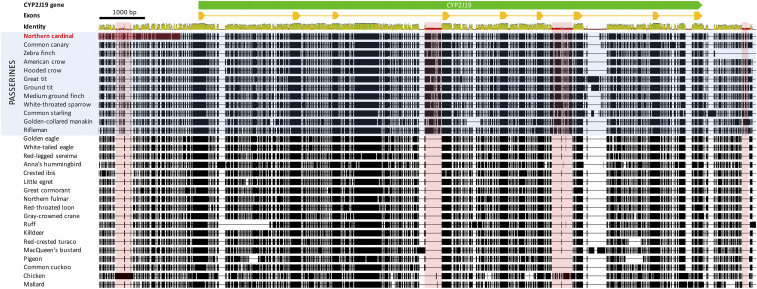
Multiple sequence alignment of the *CYP2J19* gene in birds, with red highlighted areas showing regions present in passerines but not other birds. Exons of the *CYP2J19* gene are depicted as yellow triangles. The dark red highlighted region upstream of the *CYP2J19* gene in the northern cardinal is highly disagreed with the consensus and different from other passerines (see figure 4). Passerine species included are northern cardinal (*Cardinalis cardinalis*), common canary (*Serinus canaria*), zebra finch (*Taeniopygia guttata*), American crow (*Corvus brachyrhynchos*), hooded crow (*Corvus cornix*), great tit (*Parus major*), ground tit (*Pseudopodoces humilis*), medium ground finch (*Geospiza fortis*), white-throated sparrow (*Zonotrichia albicollis*), common starling (*Sturnus vulgaris*), golden-collared manakin (*Manacus vitellinus*), and rifleman (*Acanthisitta chloris*). Non-passerine species included are golden eagle (*Aquila chrysaetos*), white-tailed eagle (*Haliaeetus albicilla*), red-legged sereima (*Cariama cristata*), Anna’s hummingbird (*Calypte anna*), crested ibis (*Nipponia nippon*), little egret (*Egretta garzetta*), great cormorant (*Phalacrocorax carbo*), northern fulmar (*Fulmarus glacialis*), red-throated loon (*Gavia stellata*), gray-crowned crane (*Balearica regulorum*), ruff (*Calidris pugnax*), killdeer (*Charadrius vociferus*), red-crested turaco (*Tauraco erythrolophus*), MacQueen’s bustard (*Chlamydotis macqueenii*), Pigeon (*Columba livia*), common cuckoo (*Cuculus canorus*), chicken (*Gallus gallus*), and mallard (*Anas platyrhynchos*).

**Figure 4:**
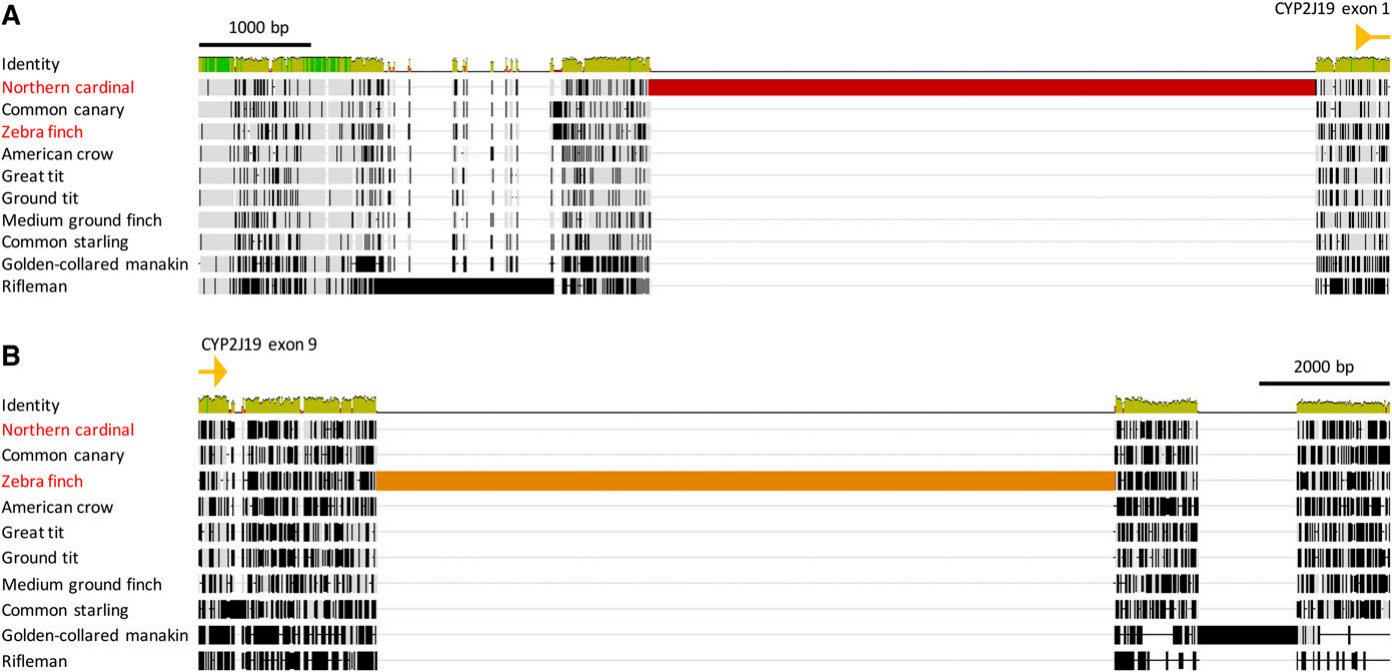
Multiple sequence alignment of (a) upstream and (b) downstream regions of the *CYP2J19* gene in passerines. A large insertion upstream of *CYP2J19* unique to the northern cardinal is highlighted in red. The insertion of a large region downstream of *CYP2J19* highlighted in orange in the zebra finch indicates the *CYP2J19B* gene in the *CYP2J2*-like cluster in this species. The names of species possessing red carotenoid coloration (*i.e.*, northern cardinal and zebra finch) are in red. Exons of the *CYP2J19* gene are depicted as yellow triangles. Refer to Fig. 3 for species names.

Two intronic insertions, as well as one non-coding region upstream and one downstream of the gene were found to be present only in passerine species (Figure 3). In addition, we identified unique insertions of large genomic regions in the two species with red carotenoid coloration out of 10 passerine species in our alignment, *i.e.*, *C. cardinalis* and *T. guttata* (Figure 4). A unique 5920 bp insertion is present 339 bp upstream of the *CYP2J19* gene in *C. cardinalis* (Figure 4a). There is a 11322 bp insertion downstream of *CYP2J19* in *T. guttata* comprising the *CYP2J19B* gene (Figure 4b; Mundy *et al.* 2016) that is not present in *C. cardinalis*. The most similar sequence to the *C. cardinalis* upstream insertion identified via BLAST (∼84% identity, 25% query cover) is a sequence annotated as “RNA-directed DNA polymerase from mobile element jockey-like” in the American crow (*Corvus brachyrhynchos*) genome assembly (Zhang *et al.* 2014), suggesting that the insertion may contain a non-long terminal repeat (non-LTR) retrotransposon (Ivanov *et al.* 1991). An endogenous retroviral insertion located upstream of the aromatase gene is proposed to be the mechanism of gene activation that lead to the henny feathers phenotype in chickens (Matsumine *et al.* 1991). The possibility that the upstream retrovirus may promote *CYP2J19* gene activation is worth further investigation.

**Figure 5:**
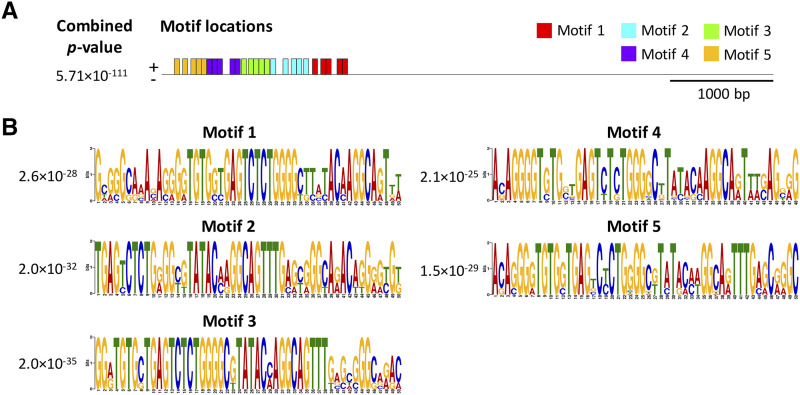
Identification of potential DNA motifs in the unique 5920 bp insertion upstream of the *CYP2J19* gene in *C. cardinalis*. (a) Locations of 25 motifs identified and their distribution in the insertion sequence. Sites on the positive (+) strand are shown above the line. Scale is shown below the sequence. (b) Logos of five motifs with statistically significant e-values provided next to the logos.

We discovered a total of 25 motifs clustered in a ∼1.7 kb region in the unique insertion sequence of *C. cardinalis* (Figure 5a). Fourteen TFs are predicted for the 5 identified motif types (Fig. S2, Table 3). No significant GO term appears to be associated with motifs 1–3 and 5, whereas significant GO terms associated with motif 3 predict molecular functions of GTP binding and hexose transmembrane transporter activity. Three predicted TFs (*i.e.*, Sp1, SREBF, and RREB1; Table 3) are associated with androgen regulation of gene expression.

**Table 3:** Transcription factors (TFs) predicted for the 5 motifs

Motif no.	TF name*^a^*	*p*-value
1	KLF13	6.32×10^−04^
	**Nfe2l2**	2.15×10^−03^
	**SP1**	3.62×10^−03^
	**SREBF1**	4.81×10^−03^
	**Ascl2 secondary**	5.18×10^−03^
2	SOX8 DBD5	3.99×10^−03^
3	**Nfe2l2**	3.72×10^−04^
	MAF NFE2	2.78×10^−03^
	**Bach1 Mafk**	3.14×10^−03^
	Zfp128 primary	3.40×10^−03^
	E2F7	5.52×10^−03^
4	**Nfe2l2**	1.05×10^−03^
	**RREB1**	2.86×10^−03^
	**SP1**	4.73×10^−03^
	**Bach1 Mafk**	4.91×10^−03^
5	**SP1**	2.04×10^−03^
	**Ascl2 secondary**	2.06×10^−03^
	**SREBF1**	2.18×10^−03^
	EBF1	3.42×10^−03^
	**Nfe2l2**	3.95×10^−03^
	KLF16	4.83×10^−03^
	SP3	4.83×10^−03^
	**RREB1**	4.96×10^−03^

a Bold names indicates TFs predicted for more than one motif.

*Cis*-regulatory elements play a crucial role in the development of sexually dimorphic traits by the integration of sex- and region-specific transcription factors (Williams and Carroll 2009). This regulatory control is proposed to be a general mechanism of sexually dimorphic gene expression and trait development (Williams and Carroll 2009; Meiklejohn *et al.* 2014). In birds, sexual dimorphism is strongly affected by steroid sex hormones (Kimball 2006). Sex hormones such as androgen may indirectly influence the expression of androgen-regulated genes, through binding to transcription factors that interact with regulatory elements such as enhancer and cause sex-biased gene expression, which leads to sex-specific phenotypes (Coyne *et al.* 2008; Mank 2009). Whereas male plumage appears to be testosterone-dependent in passerines (Kimball 2006), the molecular mechanisms controlling sex-biased gene expression and development of sexually dimorphic traits in birds is largely unknown (Kraaijeveld 2019; Gazda *et al.* 2020a). In this study, we identified potential TFs that may regulate the expression of the red plumage color gene *CYP2J19*. Many CYP450s are differentially expressed between the sexes (Rinn and Snyder 2005). Some of our TFs predicted to interact with the insertion unique to *C. cardinalis* are androgen-regulated, including Sp1, which is involved in the androgen activation of the vas deferens protein promoter in mice (Darne *et al.* 1997) and sterol regulatory element-binding factor (SREBF, aka SREBP), which is involved in androgen-regulated activation mechanisms of target genes (Heemers *et al.* 2006). Another predicted TF, the zinc finger protein Ras-responsive element binding protein (RREB1), is a co-regulator of androgen receptor (AR) and plays a role in androgenic signaling by affecting AR-dependent transcription (Mukhopadhyay *et al.* 2007). The interaction between androgens, the implicated TFs and their corresponding binding motifs may underlie the development of sexually dimorphic red plumage color in *C. cardinalis*. In addition, the TF nuclear factor erythroid-derived 2-like 2 (Nfe2l2, also known as nuclear factor erythroid 2-related factor 2, Nrf2) is predicted to bind to all but motif 2 (Figure 5). The activity of Nrf2 is influenced by changes in oxidative stress, and it regulates the expression of numerous antioxidant proteins (Schultz *et al.* 2014). Because the production of red ketacarotenoids via *CYP2J19* is sensitive to oxidative state (Lopes *et al.* 2016), this oxidation-dependent TF suggests a possible link between production of red pigments and the oxidative stress faced by the organism, which may reflect an association between red carotenoid coloration and individual quality (Hill and Johnson 2012).

The above non-exonic regions in and outside of the *CYP2J19* gene are candidates of regulatory elements responsible for modulating red carotenoid-based coloration in passerines and *C. cardinalis*. Noncoding regulatory regions may be subject to less pleiotropic constraint than protein-coding genes (Carroll *et al.* 2008), and many CNEEs act as enhancers with regulatory functions (Seki *et al.* 2017; Sackton *et al.* 2019). In particular, the large non-exonic region rich with TF-binding motifs upstream of the *C. cardinalis* *CYP2J19* gene may play a role in the strong sexual dichromatism and striking bright red male color in this species. It is also possible that this insertion is present in other Cardinalidae species and play a role in the sexual dichromatism commonly found in this family. The mechanism of plumage color development and sexual dichromatism, and the regulatory role of those genomic regions identified in this study are fruitful areas for future research.

Furthermore, bilateral gynandromorph *C. cardinalis* has been reported, which exhibited bright red color of a male on one side of the body and dull appearance of a female on another side (Peer and Motz 2014). Gynandromorphs were male:female chimeras in chicken (Zhao *et al.* 2010) and zebra finch (Agate *et al.* 2003), and were likely to arise under the same mechanism in *C. cardinalis* and other birds (Peer and Motz 2014; Brenner *et al.* 2019). The gynandromorphic phenotype was due to different responses of somatic cells with male or female origin to the same profile of circulating hormones (Zhao *et al.* 2010; Agate *et al.* 2003), and it indicates how avian somatic cells with different sex chromosomes could exhibit sex differences in gene expression in the absence of hormonal differences (Wijchers and Festenstein 2011). This inherent sex identity in avian somatic cells suggests epigenetic marks generated during early ontogeny to be the major factor underlying sexually dimorphic gene expression (Wijchers and Festenstein 2011; Rice *et al.* 2016). Epigenetic regulation is therefore likely to contribute to sensitivity of cells to sex hormones and hence sexual dichromatism in *C. cardinalis*. Facilitated by the *C. cardinalis* genome from this study, future studies on epigenetic regulations such as DNA methylation and chromatin accessibility, especially on the candidate regulatory regions of the *CYP2J19* gene, will shed light on the developmental mechanism of sexual dichromatism in this and other avian species.

### Demographic history of C. cardinalis

**Figure 6:**
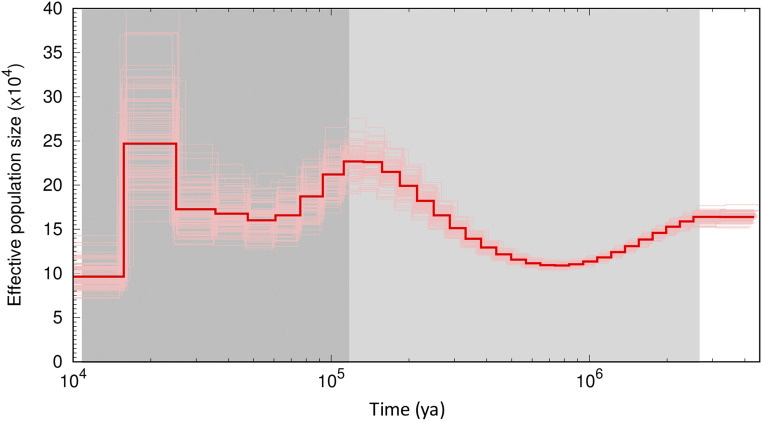
Demographic history of the northern cardinal inferred using PSMC. Bold red line is the median effective population size estimate, whereas thin lines are 100 individual bootstrap replicates. The light and dark gray areas indicate the Pleistocene epoch and Last Glacial Period, respectively.

The PSMC analysis (Figure 6) suggested a demographic history of *C. cardinalis* characterized by a fluctuation in the historic effective population size between 100,000–250,000 around ∼2,000,000 years ago (ya). The population was at ∼160,000 at the beginning of Pleistocene epoch at 2,580,000 ya, then decreased to 110,000 in ∼800,000 ya, and increased to ∼230,000 at the beginning of the Last Glacial Period around 115,000 ya. The population started to decline again after the beginning of the Last Glacial Period (LGP). The decrease in effective population size observed at the beginning of Pleistocene might be due to the divergence of *C. cardinalis* into different subspecies (Provost *et al.* 2018). The population decline after the start of the LGP is consistent with the ecological niche model that indicates a dramatic range reduction for *C. cardinalis* during the LGP (Smith *et al.* 2011). The *C. cardinalis* genome and this PSMC analysis will help facilitate more detailed analysis of demographic history and population genomics using whole-genome data from different populations and subspecies.

## Conclusion

We used small-fragment and mate-pair libraries Illumina sequencing data to generate a draft genome assembly of the northern cardinal, *C. cardinalis*. Comparative analyses revealed conserved non-exonic regions unique to the *CYP2J19* gene in passerines and in *C. cardinalis*, which may play a role in the regulation of red carotenoid-based plumage coloration. The motifs discovered in the lineage-specific upstream region of *CYP2J19* in *C. cardinalis* suggest potential *cis*-regulatory mechanisms underlying sexual dichromatism. The assembled *C. cardinalis* genome is therefore useful for studying genotype–phenotype associations and sexual dichromatism in this species. The PSMC analysis based on this genome reveals fluctuations in historic effective population size concordant with geographical events and subspecies divergence. As one of the first genome sequenced in Cardinalidae, and the highest in quality thus far, it will also be an important resource for the comparative study of plumage color evolution in passerines and birds in general.
